# Long-Term Environmental Surveillance of *Pseudomonas aeruginosa* in a Hospital Water Distribution System: Trends in Prevalence, Biofilm Formation, and Antimicrobial Susceptibility

**DOI:** 10.3390/pathogens15080868

**Published:** 2026-08-20

**Authors:** Maria Luisa Cristina, Elisa Schinca, Carolina Piccinini, Sara Cima, Gianluca Ottria, Chiara Dupont, Alessio Carbone, Marina Sartini

**Affiliations:** 1 Department of Health Sciences, University of Genoa, 16132 Genoa, Italy; elisa.schinca@unige.it (E.S.); carolina.piccinini@libero.it (C.P.); saracima.9999@gmail.com (S.C.); gianluca.ottria@unige.it (G.O.); chiara.dupont@unige.it (C.D.); 2 Operating Unit Hospital Hygiene, Galliera Hospital, 16128 Genoa, Italy; 3 Department of Medicine, Division of Medical Oncology, Galliera Hospital, 16128 Genoa, Italy; alessio.carbone@galliera.it

**Keywords:** *Pseudomonas aeruginosa*, water, healthcare settings, biofilm, antimicrobial susceptibility

## Abstract

Background: Hospital water systems are major environmental reservoirs of *Pseudomonas aeruginosa*, but evidence on the long-term impact of Water Safety Plans (WSPs) on environmental prevalence, antimicrobial resistance, and biofilm-forming ability remains limited. This study evaluated the long-term occurrence of *P. aeruginosa* in a hospital water distribution system over a 15-year surveillance period. Methods: A 15-year environmental surveillance study was conducted in a tertiary-care hospital in Northern Italy. Water samples were cultured for *P. aeruginosa*, and recovered isolates were assessed for antimicrobial susceptibility and biofilm-forming ability. Temporal trends were analysed using generalized linear and segmented logistic regression models. Results: Among 1696 water samples, the overall prevalence of *P. aeruginosa* was 2.93%, with a significant annual decline of 12% (OR 0.88, 95% CI 0.82–0.94; *p* < 0.001). Segmented regression identified a marked reduction after the fourth year (OR 0.18, 95% CI 0.06–0.55; *p* = 0.003). Over time, isolates shifted toward weak or non-biofilm-producing phenotypes while maintaining high antimicrobial susceptibility, with only one multidrug-resistant isolate detected. Conclusions: Long-term surveillance integrated within a comprehensive WSP was associated with reduced *P. aeruginosa* contamination, decreased biofilm-forming ability, and persistently low antimicrobial resistance.

## 1. Introduction

*Pseudomonas aeruginosa* is a major opportunistic pathogen and one of the leading causes of healthcare-associated infections (HAIs), particularly among immunocompromised patients and individuals admitted to intensive care units [1]. It is responsible for a broad spectrum of infections, including pneumonia, bloodstream infections, urinary tract infections, and surgical site infections, and is associated with substantial morbidity, mortality, prolonged hospitalization, and increased healthcare costs [2]. Its remarkable capacity to survive under adverse environmental conditions and to rapidly acquire antimicrobial resistance makes *P. aeruginosa* a major challenge for infection prevention and control [3,4,5].

The acquisition of *P. aeruginosa* HAIs may occur through both endogenous and exogenous routes. Endogenous infections originate from the patient’s own microbiota, whereas exogenous transmission may occur through the hands of healthcare workers, contaminated medical devices, or environmental reservoirs, particularly components of hospital water distribution systems [6,7,8]. Consequently, hospital water systems are increasingly recognized not only as environmental reservoirs but also as potential sources of patient colonization, sporadic infections, and healthcare-associated outbreaks [9,10].

Hospital water distribution systems provide favorable ecological conditions for the persistence and biofilm-mediated colonization of *Pseudomonas aeruginosa*, making them an important environmental reservoir in healthcare settings [11]. Their complex plumbing architecture, the presence of low-flow or stagnant sections, and physicochemical conditions that promote microbial adhesion facilitate biofilm development and the establishment of persistent bacterial communities [12,13].

*P. aeruginosa* may be introduced into hospital water systems through incoming source water, storage facilities, or colonized sections of the distribution network. Once established, it readily adheres to the inner surfaces of plumbing materials, where it forms biofilms that enable long-term persistence and reduce the efficacy of conventional water treatment and disinfection procedures, including chlorination [14]. As these biofilms mature, they become stable environmental reservoirs that continuously or intermittently release planktonic bacteria into the water stream, thereby contaminating taps, showerheads, sink drains, toilets, and other water outlets that have repeatedly been implicated in patient colonization and healthcare-associated infections [15,16,17,18,19]. Biofilms protect bacterial cells from environmental stressors and disinfectants and promote close bacterial interactions that facilitate horizontal gene transfer and adaptive evolution. Furthermore, a proportion of embedded cells may enter a viable but non-culturable (VBNC) state, escaping detection by conventional culture-based surveillance and potentially leading to an underestimation of environmental contamination [20].

The long-term persistence of biofilm-associated populations also creates favorable conditions for the selection and maintenance of antimicrobial-resistant *P. aeruginosa*. Environmental investigations have increasingly reported multidrug-resistant (MDR) isolates and strains harboring clinically relevant resistance determinants in water and biofilm samples [5,21]. Owing to its intrinsic resistance mechanisms, chromosomal adaptability, and capacity to acquire mobile genetic elements encoding carbapenemases and extended-spectrum β-lactamases, *P. aeruginosa* readily evolves into multidrug-resistant (MDR), extensively drug-resistant (XDR), pandrug-resistant (PDR), and difficult-to-treat resistant (DTR) phenotype, significantly limiting therapeutic options [3,22].

According to the standardized phenotypic definitions, MDR is defined as acquired non-susceptibility to at least one agent in three or more antimicrobial categories, XDR as non-susceptibility to at least one agent in all but two or fewer antimicrobial categories, and PDR as non-susceptibility to all agents in all antimicrobial categories [23]. DTR, which is based on the availability of effective first-line therapeutic options rather than on the number of antimicrobial categories affected, is defined as non-susceptibility to all first-line agents within the carbapenem, extended-spectrum cephalosporin, and fluoroquinolone classes [24].

Furthermore, internationally disseminated high-risk clonal lineages have been identified in environmental reservoirs, highlighting the potential contribution of hospital water systems to the maintenance and dissemination of clinically relevant resistant strains [25,26].

Despite the growing recognition of hospital water systems as important reservoirs of *Pseudomonas aeruginosa*, most studies have focused on outbreak settings or short-term environmental surveys. Consequently, evidence on the long-term evolution of environmental contamination following the implementation of Water Safety Plans (WSPs)—the risk management approach recommended by the World Health Organization [27] for drinking-water systems—remains limited, particularly regarding temporal changes in antimicrobial susceptibility and biofilm-forming ability of environmental isolates. Longitudinal studies extending beyond a decade are especially scarce.

Therefore, the present study aimed to characterize the long-term environmental occurrence of *Pseudomonas aeruginosa* in a hospital water distribution system through a 15-year surveillance program. Specifically, we evaluated temporal trends in environmental prevalence, antimicrobial susceptibility patterns, and biofilm-forming ability of environmental isolates to assess how the implementation of continuous water management strategies was associated with changes in these microbiological characteristics over time.

## 2. Materials and Methods

### 2.1. Setting

The study was conducted at a nationally renowned tertiary-care hospital in northern Italy, organized by treatment intensity levels. The facility, which has a total of 458 beds, is composed of eight separate pavilions built at the end of the 19th century and two pavilions constructed in the second half of the 20th century, housing the operating theatres, the intensive care unit, the neonatal unit, the Emergency Department (ED) and a number of inpatient wards.

Due to the structural characteristics of the building and the age of a substantial portion of the hospital water distribution system, continuous disinfection with sodium hypochlorite was adopted as the most appropriate water treatment strategy.

### 2.2. Water Sampling and Microbiological Analysis

Water samples were collected from showers, taps located in both high-risk and non-high-risk hospital wards, and from storage tanks by direct immersion. For tap sampling, any existing flow straightener was first removed, and the outlet was flushed by allowing the water to run for 1–3 min before sample collection.

Conversely, at outlets fitted with point-of-use (POU) absolute filters, water samples were collected directly from the filter outlet without removing the filtration device.

At each site, samples were gathered utilizing sterile, single-use polyethylene bottles pre-treated with sodium thiosulphate to neutralize residual chlorine in the sampled water. All containers were immediately placed in refrigerated, thermally insulated transport boxes and processed in the laboratory within two hours of arrival.

The samples were analysed for the detection of *Pseudomonas aeruginosa* using a standard method based on the filtration technique (UNI EN ISO 16266) [28]. Briefly, the water sample (100 mL) was filtered through a cellulose ester membrane (0.45 µm porosity, 47 mm diameter); the membrane was then placed on Pseudomonas CN agar (Liofilchem, Roseto degli Abruzzi (TE), Italy), which is a selective medium for *P. aeruginosa*, and subsequently cultured at 36 ± 2 °C for 44 ± 4 h before colony counting.

Definitive identification of *P. aeruginosa* was assigned to colonies exhibiting a distinctive blue/green pyocyanin pigmentation. Conversely, colonies displaying fluorescence without pyocyanin production, or those with a reddish-brown appearance, were classified as presumptive isolates; these were subsequently verified using MALDI-TOF mass spectrometry.

Upon collection, all strains were immediately transferred into Clearstable vials (Biosigma, Cona, Venezia, Italia) and systematically frozen for long-term storage at −80 °C.

The *P. aeruginosa* isolates were gradually frozen at −80 °C as they were collected, and then simultaneously revitalized and tested for biofilm and antibiotic resistance in 2026.

### 2.3. Biofilm

Biofilm formation was assessed using a microplate crystal violet assay. The bacterial isolates were cultured on blood agar at 37 °C for 24 h; individual colonies were inoculated into 2 mL of Tryptic Soy Broth (TSB) and incubated overnight at 37 °C.

The cultures were standardized to 1 McFarland and diluted 1:100 in TSB supplemented with 0.5% glucose. Aliquots of 200 µL were dispensed into sterile 96-well microplates. Negative controls (sterile medium) were included in each plate.

The plates were incubated under static conditions at 37 °C for 24 h. At the end of incubation, the bacterial suspension was removed, and the wells were washed three times with sterile water. After air-drying, the adherent biofilms were stained with 0.5% crystal violet for 15 min at room temperature. Excess dye was removed by washing with distilled and sterile water, and the plates were left to dry completely.

The bound dye was solubilized with 20% ethanol, and the optical density (OD) was measured at 570 nm using a microplate reader (Epoch).

To determine the background, the OD values of the negative controls from all plates were pooled, given their high homogeneity, to calculate a global mean and the corresponding standard deviation, thereby minimizing inter-plate variability. The cut-off value (ODc) was defined as: ODc = media_blank + 3 × SD_blank.

Biofilm formation was then classified according to the criteria of Stepanović et al. [29] as follows:

- Non-producer: OD ≤ ODc.
- Weak producer: ODc < OD ≤ 2 × ODc.
- Moderate producer: 2 × ODc < OD ≤ 4 × ODc.
- Strong producer: OD > 4 × ODc.

### 2.4. Antibiotic Susceptibility Testing

The Antimicrobial Susceptibility Testing (AST) was carried out on fresh colonies (within 24 h of plating) using the VITEK^®^ 2 COMPACT system (bioMérieux, Marcy-l’Étoile, France) with the AST-N440 card, which included Piperacillin/Tazobactam, Ceftazidime, Ceftazidime/Avibactam, Ceftolozane/Tazobactam, Cefepime, Aztreonam, Imipenem, Meropenem, Imipenem/Relebactam, Tobramycin, Ciprofloxacin, and Colistin. These antibiotics correspond to the panel recommended by the current version of the EUCAST breakpoint tables for *Pseudomonas* spp., which include compounds such as Meropenem, Ceftazidime/Avibactam, Ceftolozane/Tazobactam and Imipenem/Relebactam, specifically indicated for *Pseudomonas aeruginosa* [30].

### 2.5. Statistical Analysis

All characteristics were expressed as absolute values and percentages. The Chi-square test was used to assess independence between variables. Temporal trends in *Pseudomonas aeruginosa* positivity were evaluated using a generalized linear model (GLM) with a binomial distribution and logit link function, based on the number of positive samples and the total number of samples collected. Calendar year was entered as a continuous predictor, and sampling site (high-risk units, wards, and storage tanks) was included as a fixed effect to account for differences in the distribution of samples across sampling sites. Results are reported as odds ratios (ORs) with 95% confidence intervals (95% CIs). Because evidence of overdispersion was observed, standard errors were scaled using the Pearson χ^2^-based dispersion parameter.

Potential heterogeneity in temporal trends among sampling sites was assessed by including a year-by-sampling-site interaction in the model. The overall significance of the interaction was evaluated using a Wald test. Site-specific model-predicted probabilities of positivity and corresponding 95% CIs were estimated and graphically displayed together with the observed proportions.

To explore potential changes in the temporal pattern, segmented logistic regression models with candidate breakpoints were compared using the Akaike Information Criterion (AIC), and nested models were evaluated by likelihood ratio tests. Year 5 was identified as the optimal breakpoint and was subsequently used in the segmented analysis. The segmented model included terms for the pre-breakpoint temporal trend, the immediate level change at year 5, and the change in slope after year 5, and was adjusted for sampling site. Standard errors were scaled using the Pearson χ^2^-based dispersion parameter.

Because unfiltered sampling points in high-risk units progressively decreased during the surveillance period as outlets were converted to filtered outlets, a sensitivity analysis was performed excluding high-risk units. The same segmented model was fitted to data from wards and storage tanks to assess the robustness of the level and slope changes observed at year 5. The post-breakpoint temporal trend was estimated as the linear combination of the pre-breakpoint slope and the change in slope.

A two-sided *p*-value < 0.05 was considered statistically significant. All statistical analyses were performed using Stata/SE 19.5 (StataCorp LLC, College Station, TX, USA).

## 3. Results

During the study period (2012–2026), a total of 1696 water samples were collected from general patient care wards, high-risk hospital units (including Operating Rooms, Intensive Care Unit, and Neonatology Unit), and storage tanks.

During the 15-year surveillance period, the overall prevalence of *Pseudomonas aeruginosa* was 2.93%, with annual values ranging from 0% in the final year of surveillance to a peak of 11.22% in the fourth year. Stratification by sampling site showed that the highest positivity rate was detected in water samples collected from storage tanks (Table 1).

Following the fourth year of water quality monitoring, the hospital implemented a series of measures aimed at improving the microbiological quality of the water distribution system. To support this process, a multidisciplinary team was established and developed a Water Safety Plan (WSP). Among the measures identified and implemented, in addition to continuous water disinfection, were several preventive and risk reduction actions, including daily flushing of outlets that were not regularly or only infrequently used to prevent water stagnation and reduce the risk of microbial contamination.

Additionally, there was also annual replacement of flow straighteners, or more frequent replacement when visibly scaled or encrusted, with replacement being preferred to removal, to minimize water splashing and the potential dissemination of microorganisms, and the installation of point-of-use (POU) filters in high-risk hospital units, including Intensive Care Units (ICUs), Neonatology, and Operating Rooms.

After adjustment for sampling site and overdispersion, *Pseudomonas aeruginosa* positivity showed a significant downward temporal trend, with an approximately 11% annual reduction in the odds of positivity (OR = 0.89, 95% CI 0.80–0.99; *p* = 0.029) (Table 2).

**Figure 1:**
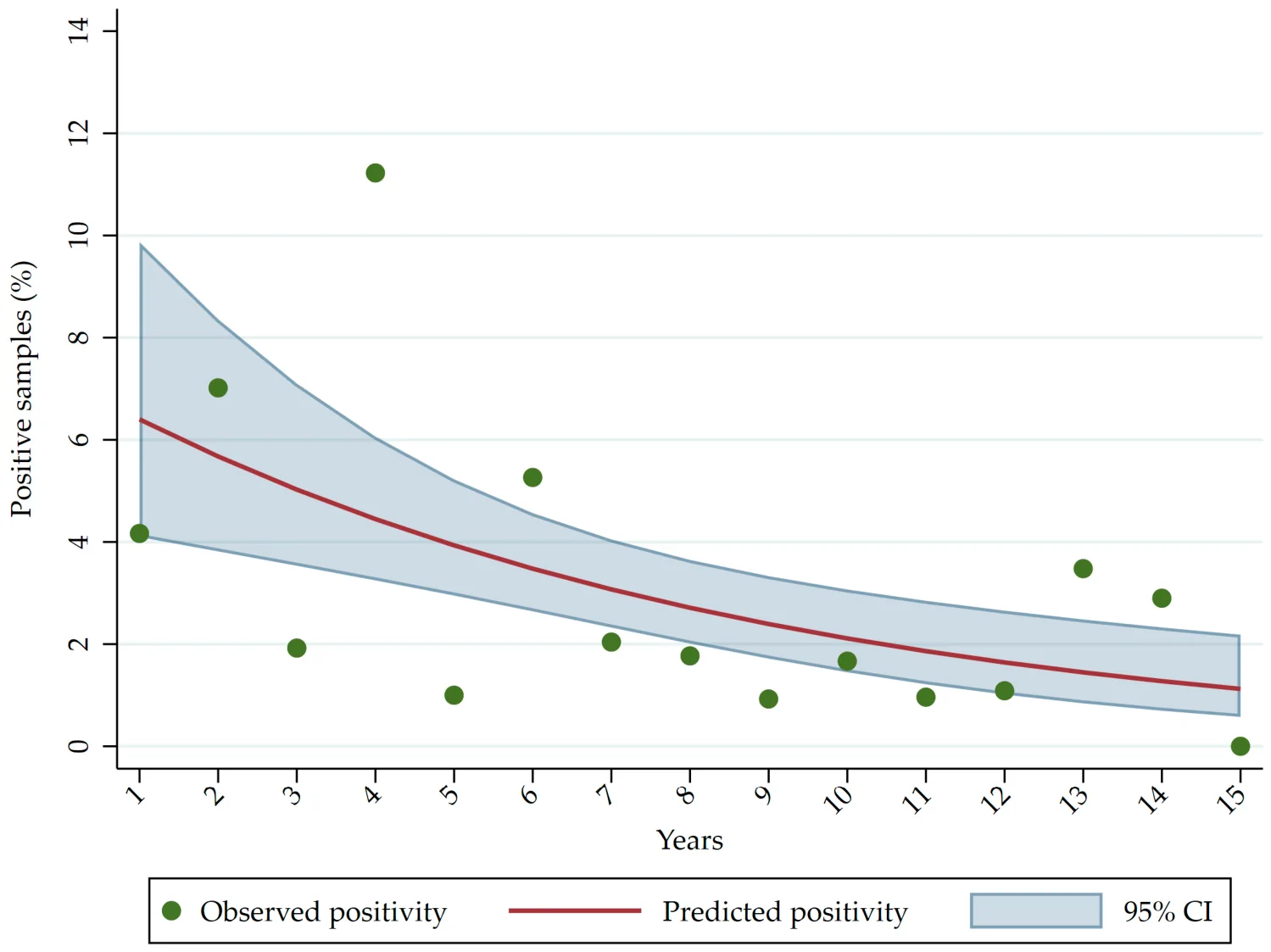
Observed positivity probability to detect *Pseudomonas aeruginosa* over the surveillance period. The solid line represents the probabilities estimated by the logistic regression model, while the shaded area indicates the corresponding 95% confidence interval.

To assess whether this trend differed among sampling sites, a year-by-sampling-site interaction was tested. Although site-specific patterns differed descriptively the overall interaction was not statistically significant (Wald χ^2^(2) = 2.95; *p* = 0.229), providing no evidence of significant heterogeneity in temporal trends among high-risk units, wards, and storage tanks (Figure 1).

The temporal pattern was further investigated using segmented binomial logistic regression. Year 5, previously identified as the optimal breakpoint by comparison of candidate models, was retained in the adjusted analysis. No significant temporal trend was observed before year 5 (OR = 1.38, 95% CI 0.87–2.18; *p* = 0.168). At year 5, however, the odds of positivity decreased by approximately 84% (OR = 0.16, 95% CI 0.04–0.68; *p* = 0.013). The subsequent change in slope was not statistically significant (OR = 0.70, 95% CI 0.43–1.13; *p* = 0.143) (Table 3).

Because unfiltered sampling points in high-risk units progressively decreased as outlets were converted to filtered outlets, a sensitivity analysis was performed excluding high-risk units. Among wards and storage tanks, positivity increased significantly before year 5 (OR per year = 2.84, 95% CI 1.18–6.83; *p* = 0.020), followed by a marked immediate reduction at year 5 (OR = 0.07, 95% CI 0.01–0.36; *p* = 0.002) and a significant change in slope (OR = 0.33, 95% CI 0.14–0.82; *p* = 0.016). Thereafter, no significant temporal trend was observed (OR per year = 0.95, 95% CI 0.81–1.12; *p* = 0.537) (Table 4). Thus, the level change at year 5 persisted after exclusion of high-risk units.

Among the positive water samples, the mean *P. aeruginosa* concentration over the 15-year surveillance period was 126.23 CFU/100 mL (SD 181.87, median 37 CFU/100 mL, range 1–744 CFU/100 mL).

According to the classification proposed by NHS England [31], *Pseudomonas aeruginosa* concentrations were divided into two categories: 1–10 CFU/100 mL and >10 CFU/100 mL. Based on this classification, over the 15-year surveillance period, 54.0% of positive samples had concentrations ranging from 1 to 10 CFU/100 mL, whereas 46.0% had concentrations >10 CFU/100 mL.

**Figure 2:**
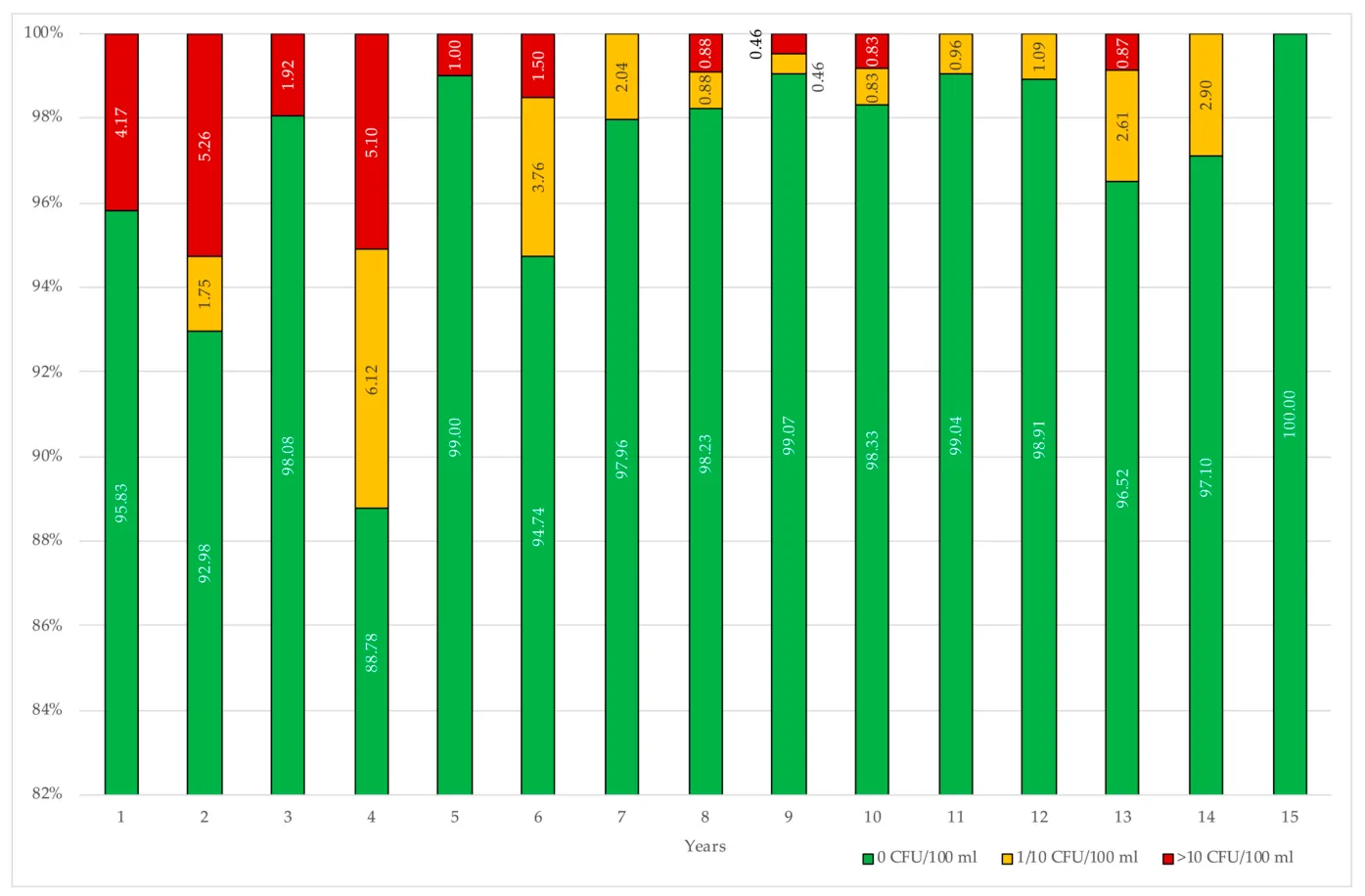
Percentage distribution of *P. aeruginosa* according to the classification proposed by NHS.

Figure 2 shows the percentages of water samples that were negative for *P. aeruginosa*, positive with concentrations ranging from 1 to 10 CFU/100 mL, and positive with concentrations >10 CFU/100 mL. To ensure that even very small percentages were clearly visible, the y-axis was truncated and set to start at 82%.

Regarding the evaluation of chemical parameters, residual chlorine levels were below the limit of detection in 67.74% of water samples positive for *P. aeruginosa*.

### 3.1. Biofilm

The isolates were subsequently assessed for biofilm-forming ability and classified as non-producers or, when positive, as weak, moderate, or strong biofilm producers.

**Figure 3:**
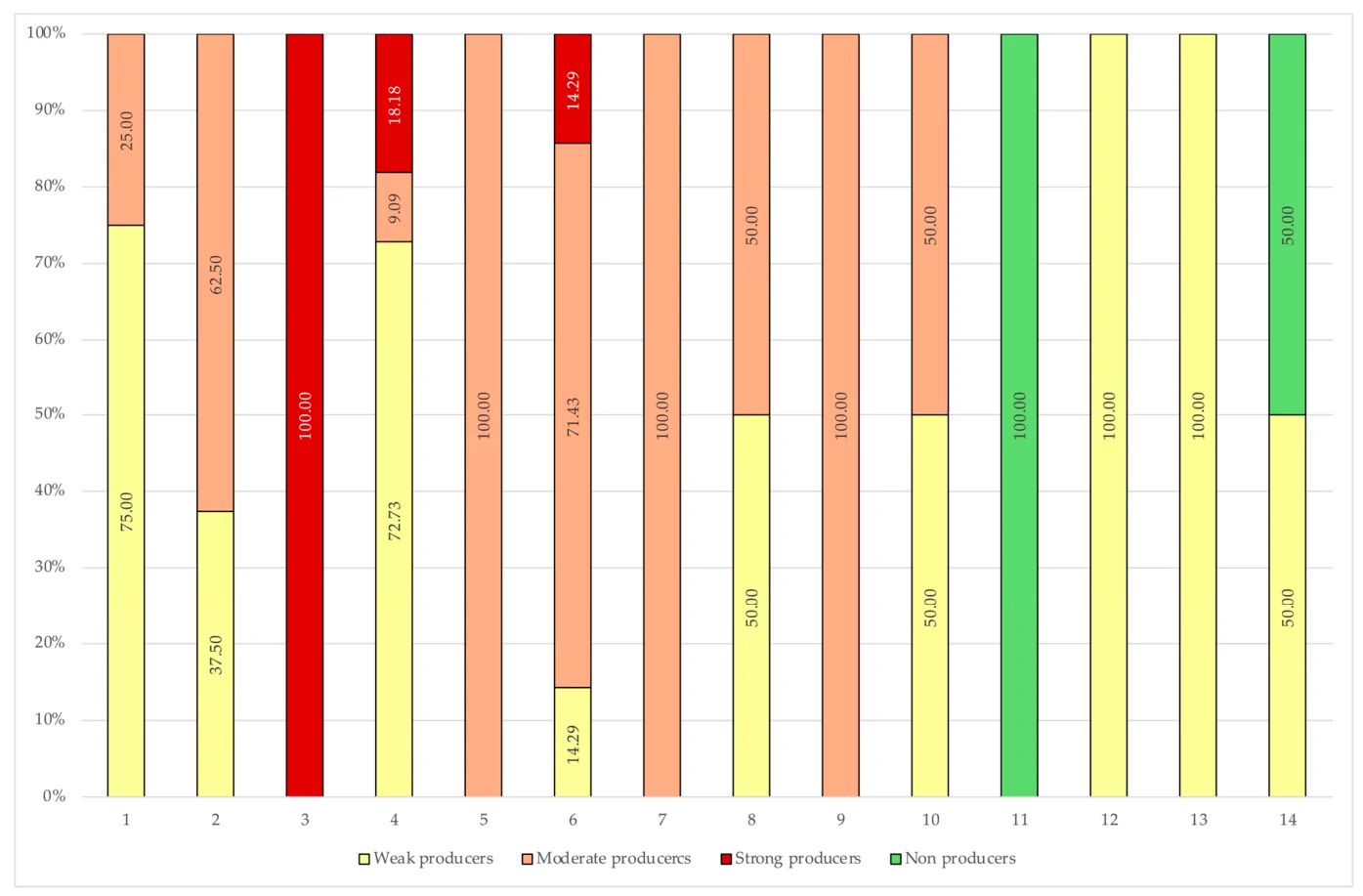
Temporal distribution of *Pseudomonas aeruginosa* according to biofilm-forming capacity.

Figure 3 illustrates the temporal distribution of *P. aeruginosa* isolates according to their biofilm-forming ability. Considerable variability was observed over the surveillance period. During the first years, most isolates were classified as moderate biofilm producers, although weak and strong biofilm producers were also detected. Strong biofilm-producing isolates were identified only during the early years of surveillance (years 3, 4, and 6), with year 3 characterized exclusively by strong biofilm producers. In the following years, moderate biofilm producers predominated, whereas strong biofilm producers were no longer detected. From year 11 onwards, a progressive shift toward weak or non-biofilm-producing isolates was observed. Notably, all isolates recovered in year 11 were classified as non-biofilm producers, while all isolates collected in years 12 and 13 were weak biofilm producers. In the final year of surveillance, weak and non-biofilm producers accounted for equal proportions of the isolates (50% each).

### 3.2. Antimicrobial Susceptibility Testing

**Figure 4:**
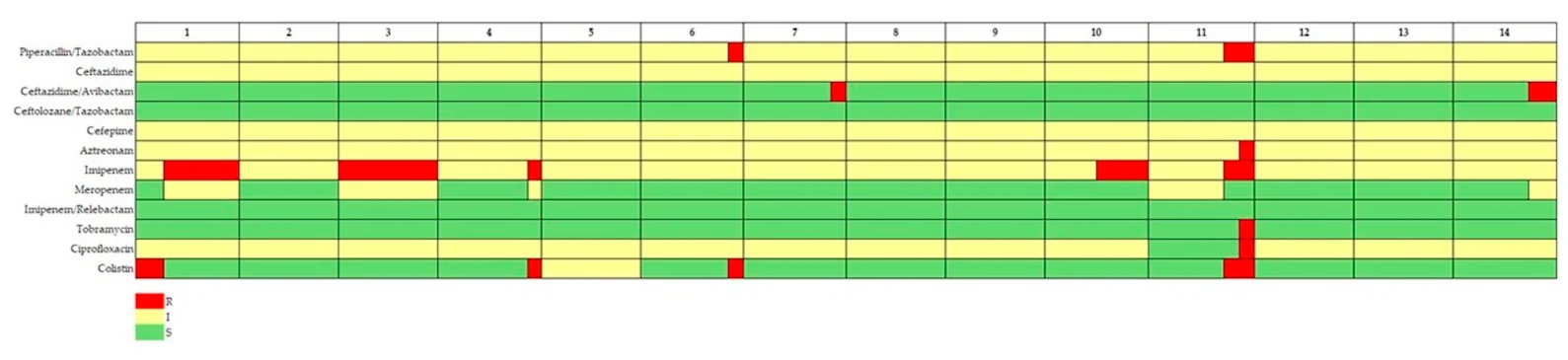
Temporal distribution of *P. aeruginosa* antimicrobial spectrum.

The antimicrobial susceptibility of the environmental *P. aeruginosa* isolates was subsequently assessed, and temporal changes in resistance patterns over the 15-year surveillance period are shown in Figure 4. The proportion of susceptible (green), intermediate (yellow), and resistant (red) isolates for each antimicrobial agent is represented by the relative size of the corresponding-coloured segments. Overall, the isolates remained highly susceptible throughout the surveillance period. Intermediate susceptibility was observed more frequently than resistance for several antimicrobial agents, whereas resistant isolates were detected only sporadically. Resistance was mainly observed against imipenem during the early years of surveillance and reappeared in year 10, while isolated resistant phenotypes to Piperacillin/Tazobactam, Aztreonam, Tobramycin, Ciprofloxacin, and Colistin were detected only in individual years. No resistance was observed to Meropenem, Imipenem/Relebactam, or Ceftolozane/Tazobactam.

According to the international definition of multidrug resistance (MDR), none of the *P. aeruginosa* isolates collected during the surveillance period were classified as MDR, except for one isolate, which showed resistance to the following antibiotics: Piperacillin/Tazobactam, Aztreonam, Imipenem, Tobramycin, Ciprofloxacin, and Colistin.

## 4. Discussion

To our knowledge, this study represents one of the longest longitudinal investigations of *P. aeruginosa* in a hospital water distribution system and one of the few to evaluate temporal changes before and after the implementation of a Water Safety Plan over a 15-year environmental surveillance period.

Over the 15-year surveillance period, the overall prevalence of *P. aeruginosa* remained low (2.93%) and progressively declined, reaching complete absence of positive samples in the final year of surveillance. This finding is particularly relevant considering the well-established ability of *P. aeruginosa* to persist within hospital plumbing systems through biofilm formation, making eradication challenging once colonization has occurred [13,14,32,33].

A significant reduction in positivity was observed after implementation of the Water Safety Plan (WSP), with segmented logistic regression identifying a marked decrease immediately after its introduction. Although the observational design does not allow causal inference, these findings are consistent with previous reports demonstrating that structured Water Safety Plans represent an effective risk management approach for reducing waterborne pathogens in healthcare facilities [7,27,34]. The combination of continuous disinfection, routine flushing, replacement of flow straighteners, and installation of point-of-use filters probably contributed to limiting bacterial persistence within the distribution system.

The highest positivity rates were consistently observed in storage tanks. This observation agrees with previous studies identifying water storage systems as critical ecological niches because of increased water age, reduced disinfectant residuals, sediment accumulation and enhanced biofilm development [14,35,36]. Storage tanks therefore represent priority sites for microbiological surveillance and preventive maintenance within hospital Water Safety Plans.

The observation that residual chlorine was below the detection limit in nearly 68% of positive samples further supports the importance of maintaining an adequate disinfectant residual throughout the distribution network. Previous investigations have demonstrated that decreasing chlorine concentrations facilitate *P. aeruginosa* survival and biofilm persistence, although biofilm-associated cells may remain protected even in chlorinated systems [11,14,36].

One of the most interesting findings of the present study concerns the temporal evolution of biofilm-forming ability. During the first years of surveillance, moderate and strong biofilm-producing isolates predominated, whereas in the later years weak or non-biofilm-producing isolates became increasingly frequent, and no strong biofilm producers were detected after year 6. Although the number of isolates was limited, this temporal shift may suggest that continuous environmental management progressively reduced the persistence of highly adapted environmental clones.

Biofilm formation represents one of the principal mechanisms allowing *P. aeruginosa* to colonize hospital water systems, protecting bacterial cells from disinfectants and environmental stresses while facilitating long-term persistence [14,33,37]. Therefore, the reduction in isolates exhibiting a strong biofilm phenotype may represent an indirect indicator of improved ecological control within the water distribution system.

Despite prolonged persistence within the hospital water environment, antimicrobial susceptibility remained remarkably stable throughout the study period. Only sporadic resistance to individual antimicrobial agents was observed, and a single multidrug-resistant isolate was identified. Moreover, complete susceptibility was maintained against the newer β-lactam/β-lactamase inhibitor combinations Ceftolozane/Tazobactam and Imipenem/Relebactam.

These findings contrast with the widespread assumption that environmental reservoirs necessarily constitute important sources of multidrug-resistant *P. aeruginosa*. Rather, they suggest that the hospital water distribution system primarily functions as an ecological reservoir in which bacterial persistence depends mainly on adaptation to environmental conditions and biofilm formation rather than on antibiotic selective pressure [12].

Similar observations have been reported in environmental studies showing that adaptation to plumbing systems is driven predominantly by ecological rather than clinical selective pressures [2,3,38].

This study has some limitations. First, it was conducted in a single hospital, which may limit the generalizability of the findings to other healthcare settings with different water distribution systems and management practices. Second, although the implementation of the Water Safety Plan was associated with a significant reduction in *P. aeruginosa* positivity, the observational design does not allow causal relationships to be established. Finally, molecular typing of environmental isolates was not performed, preventing assessment of the genetic relatedness and long-term persistence of specific *P. aeruginosa* clones within the hospital water distribution system.

Furthermore, it was not possible to reliably assess whether the progressive annual decline in *P. aeruginosa* detection rates within the hospital water reservoir was associated with a corresponding reduction in the incidence of clinical *P. aeruginosa* infections. Over the 15-year observation period, changes in the hospital information systems resulted in heterogeneous data collection and storage procedures, precluding the availability of a standardized longitudinal database and, consequently, robust comparisons of clinical infection rates over time. Such an analysis would have been particularly valuable for assessing the potential contribution of waterborne *P. aeruginosa* to the burden of clinical infections and for further elucidating the role of the hospital water distribution system as a potential reservoir for healthcare-associated infections.

Despite these limitations, this study provides one of the longest longitudinal evaluations of *P. aeruginosa* in a hospital water distribution system currently available. The 15-year surveillance period, combined with the implementation of a structured Water Safety Plan and the integrated assessment of environmental prevalence, biofilm-forming ability, and antimicrobial susceptibility, offers a comprehensive overview of the long-term ecological evolution of *P. aeruginosa* within a healthcare water network.

## 5. Conclusions

Overall, our findings suggest that long-term environmental surveillance integrated within a comprehensive Water Safety Plan can substantially reduce *P. aeruginosa* contamination while maintaining a stable environmental population characterized by decreasing biofilm-forming ability and limited antimicrobial resistance. These results reinforce current WHO recommendations advocating Water Safety Plans as the cornerstone of hospital water risk management and highlight the importance of continuous surveillance in assessing the long-term effectiveness of preventive interventions.

## Figures and Tables

**Table 1:** Percentages of samples positive for *P. aeruginosa* over the years and by sampling site.

	High-Risk Units		Wards		Storage Tanks	
Years	A	B	A	B	A	B
1	31.25 (30)	4.17 (4)	20.83 (20)	0.00	47.92 (46)	0.00
2	43.86 (50)	5.26 (6)	15.79 (18)	0.88 (1)	40.35 (46)	0.88 (1)
3	38.46 (20)	0.00	19.23 (10)	1.92 (1)	42.31 (22)	0.00
4	18.37 (18)	1.02 (1)	19.39 (19)	2.04 (2)	62.24 (61)	8.16 (8)
5	14.00 (14)	0.00	42.00 (42)	1.00 (1)	44.00 (44)	0.00
6	10.53 (14)	0.00	56.39 (75)	3.01 (4)	33.08 (44)	2.26 (3)
7	10.20 (10)	0.00	46.94 (46)	2.04 (2)	42.86 (42)	0.00
8	8.85 (10)	0.00	53.98 (61)	0.00	37.17 (42)	1.77 (2)
9	0.00 (0)	0.00	100.00 (216)	0.93 (2)	0.00	0.00
10	10.00 (12)	0.00	55.83 (67)	1.67 (2)	34.17 (41)	0.00
11	17.31 (18)	0.00	46.15 (48)	0.96 (1)	36.54 (38)	0.00
12	6.52 (6)	0.00	52.17 (48)	1.09 (1)	41.30 (38)	0.00
13	3.48 (4)	0.00	63.48 (73)	3.48 (4)	33.04 (38)	0.00
14	2.90 (4)	0.00	69.57 (96)	0.72 (1)	27.54 (38)	2.17 (3)
15	0.00	0.00	74.24 (49)	0.00	25.76 (17)	0.00

A: Total samples; B: Positive samples.

**Table 2:** Overall temporal trend in *Pseudomonas aeruginosa* positivity and assessment of heterogeneity by sampling site.

Parameter	OR	95% CI	*p*
Temporal trend, adjusted for sampling site (per year increase)	0.89	0.80–0.99	0.029
Wards vs high-risk units	0.76	0.26–2.19	0.611
Storage tanks vs high-risk units	0.72	0.26–2.02	0.535
Year × sampling site interaction, global test	—	—	0.229

OR, odds ratio; CI, confidence interval. Estimates were obtained from a binomial logistic regression model adjusted for sampling site, with standard errors scaled using the Pearson χ^2^-based dispersion parameter. Heterogeneity in temporal trends across sampling sites was assessed using the overall Wald test for the year-by-sampling-site interaction.

**Table 3:** Segmented logistic regression analysis of temporal changes in *Pseudomonas aeruginosa* positivity.

Parameter	OR	95% CI	*p*
Pre-breakpoint temporal trend (per year increase)	1.38	0.87–2.18	0.168
Immediate level change at year 5	0.16	0.04–0.68	0.013
Change in slope after year 5	0.70	0.43–1.13	0.143

OR, odds ratio; CI, confidence interval. Estimates were obtained from a segmented binomial logistic regression model adjusted for sampling site and overdispersion. The level-change estimate represents the immediate change in odds at year 5, while the slope-change estimate represents the change relative to the pre-breakpoint temporal slope.

**Table 4:** Sensitivity analysis of temporal changes in *Pseudomonas aeruginosa* positivity excluding high-risk units.

Parameter	OR	95% CI	*p*
Pre-breakpoint temporal trend (per year increase)	2.84	1.18–6.83	0.020
Immediate level change at year 5	0.07	0.01–0.36	0.002
Change in slope after year 5	0.33	0.14–0.82	0.016
Post-breakpoint temporal trend (per year increase)	0.95	0.81–1.12	0.537

OR, odds ratio; CI, confidence interval. The sensitivity analysis excluded high-risk units because unfiltered outlets in these units were progressively converted to filtered outlets. The post-breakpoint trend was estimated as the linear combination of the pre-breakpoint slope and the change in slope.

## Data Availability

The raw data supporting the conclusions of this article will be made available by the authors on request.
